# Prediction Models Versus Fibrosis-4 Index (FIB-4) for Advanced Fibrosis in Metabolic Dysfunction-Associated Steatotic Liver Disease (MASLD): A Verification-Audited Systematic Review and Meta-Analysis

**DOI:** 10.7759/cureus.117908

**Published:** 2026-10-14

**Authors:** Syed Muhammad Hassan Bukhari, Ushna Shahid, Wahhaj Qayyum, Aminah Amjad, Salwa Qayyum, Abdul Aleem

**Affiliations:** 1 Internal Medicine, South Tees Hospitals NHS Foundation Trust, Middlesbrough, GBR; 2 Internal Medicine, King's College Hospital, London, GBR; 3 Internal Medicine, James Cook University Hospital, Middlesbrough, GBR; 4 Internal Medicine, Combined Military Hospital (CMH) Lahore Medical College, National University of Medical Sciences (NUMS), Lahore, PAK; 5 Internal Medicine, Akhtar Saeed Medical & Dental College, Lahore, PAK; 6 Gastroenterology and Hepatology, County Durham and Darlington NHS Foundation Trust, Darlington, GBR

**Keywords:** advanced liver fibrosis, artificial intelligence, diagnostic accuracy, fib-4, machine learning, masld, meta-analysis, nafld, prediction models

## Abstract

Researchers increasingly propose data-driven prediction models for non-invasive fibrosis assessment in metabolic dysfunction-associated steatotic liver disease (MASLD). However, strong predictive performance alone does not show whether a model performs better than Fibrosis-4 Index (FIB-4), a simple and widely used fibrosis score based on routinely available clinical data. This review evaluated whether prediction models provide greater diagnostic discrimination than FIB-4 when both are assessed in the same patients for advanced fibrosis (≥F3). PubMed, Europe PMC, OpenAlex, and Semantic Scholar were searched for studies evaluating prediction models and conventional non-invasive fibrosis tests in adults with MASLD, MASH, NAFLD, or NASH. Eligible head-to-head comparisons required the model and FIB-4 to be evaluated in the same cohort for the same ≥F3 endpoint with verifiable test-specific areas under the receiver operating characteristic curve (AUROCs). We used automation-based screening and quantitative validation, followed by full-text verification, targeted evidence recovery, and final cohort-level adjudication. Separate-cohort biopsy-validation evidence formed the primary analysis, while internal holdout evidence was included secondarily. The effect measure was the within-study difference in AUROC (ΔAUROC), pooled using random-effects restricted maximum likelihood with Hartung-Knapp confidence intervals (CIs). Of 23,420 deduplicated records, 2,892 reports were assessed for eligibility, and 44 potential same-cohort comparisons were identified. Final verification produced three eligible head-to-head studies. The primary separate-cohort evidence comprised two studies, with study-level ΔAUROCs of 0.053 and 0.140, respectively. The pooled primary ΔAUROC was 0.086 (95% Hartung-Knapp CI -0.448 to 0.621). Adding the internal holdout study, which reported a ΔAUROC of 0.165, increased the pooled estimate to 0.126 (95% CI -0.023 to 0.276). All three point estimates were positive, but both pooled CIs included no difference. A 500-record reference assessment of automated exclusions identified no false negatives, corresponding to a negative predictive value of 100.0% (95% CI 99.2%-100.0%). The available head-to-head evidence is too sparse and imprecise to establish that prediction models outperform FIB-4. Moreover, all included evaluations were conducted by the original model-development teams rather than by independent third-party investigators. The findings should therefore be considered preliminary and do not support replacing FIB-4. Future studies should prioritise third-party multicentre validation, calibration, clinically actionable thresholds, and direct assessment of net clinical benefit.

## Introduction and background

Metabolic dysfunction-associated steatotic liver disease (MASLD) is the preferred term for what was previously known as non-alcoholic fatty liver disease (NAFLD). The revised terminology better reflects the metabolic basis of the condition and addresses limitations associated with the earlier name [1]. MASLD is closely associated with obesity, type 2 diabetes (T2D), dyslipidaemia, insulin resistance, and other cardiometabolic risk factors. As these conditions become increasingly common worldwide, MASLD has emerged as a leading cause of chronic liver disease and an important public health concern [2,3].

The clinical importance of MASLD extends beyond hepatic fat accumulation. The fibrosis stage is one of the strongest predictors of long-term outcomes, with advanced fibrosis associated with a higher risk of cirrhosis, hepatocellular carcinoma, liver-related mortality, and overall mortality [4,5]. Early identification of patients with advanced fibrosis is therefore central to clinical management. The practical challenge is to identify those who require further investigation or specialist referral while avoiding unnecessary invasive procedures in patients at low risk.

Liver biopsy remains an important reference standard for fibrosis staging, but its invasive nature, cost, sampling variability, and limited suitability for large-scale assessment limit its routine use. Clinical pathways therefore increasingly rely on non-invasive tests. Blood-based scores such as the Fibrosis-4 Index (FIB-4) and NAFLD Fibrosis Score (NFS) are widely used because they are inexpensive and based on routinely available clinical data. FIB-4 is calculated using age, aspartate aminotransferase, alanine aminotransferase, and platelet count [6,7]. The current European Association for the Study of the Liver-European Association for the Study of Diabetes-European Association for the Study of Obesity (EASL-EASD-EASO) guidance recommends non-invasive fibrosis assessment, particularly in people with T2D, obesity, cardiometabolic risk factors, abnormal liver enzymes, or radiological evidence of steatosis [8].

Despite its practical value, FIB-4 has recognised limitations. Because age is included in the score, specificity decreases in older adults, particularly those aged ≥65 years, which can increase false-positive classifications. Its diagnostic performance can also be less reliable in people with T2D, a clinically important group in MASLD [9]. Imaging-based approaches, including transient elastography and magnetic resonance elastography, can improve fibrosis assessment, but their use may be limited by cost, availability, operator dependence, body habitus, and access in primary-care or limited-resource settings [10]. These limitations have encouraged the development of more flexible prediction approaches that combine clinical, laboratory, imaging, metabolic, and demographic information.

Data-driven prediction models, including machine learning (ML), artificial intelligence (AI), and multivariable regression approaches, may improve non-invasive fibrosis assessment by identifying relationships that are not fully represented by fixed scoring systems such as FIB-4. In MASLD, such models have been developed using routine laboratory variables, electronic health records, imaging features, radiomics, histology, and multimodal data [11,12]. However, strong performance in a development dataset does not by itself show that a model will generalise well or provide useful added value in clinical practice.

Clinical adoption requires more than a high area under the receiver operating characteristic curve (AUROC). A clinically useful model should perform well in separate populations, provide adequately calibrated predictions, support clinically meaningful thresholds, and add value to existing diagnostic pathways. Reporting of these features remains inconsistent across the medical AI and prediction-model literature, particularly for external validation, calibration, subgroup performance, explainability, and clinical utility [13,14].

Previous systematic reviews have reported promising diagnostic performance for AI and ML models in MASLD-related and NAFLD-related fibrosis [11,12]. However, these reviews primarily summarised model performance across heterogeneous studies and did not specifically restrict the evidence to same-cohort head-to-head comparisons with FIB-4. As a result, differences in patient characteristics, fibrosis prevalence, reference standards, disease thresholds, and validation design may influence cross-study comparisons. Direct comparison of a prediction model and FIB-4 in the same cohort therefore provides a more clinically meaningful assessment of whether the model offers additional discrimination.

Accordingly, this systematic review and exploratory meta-analysis (MA) evaluated head-to-head differences in diagnostic discrimination between individual data-driven prediction models and FIB-4 when both were assessed in the same cohort for advanced fibrosis (≥F3) in adults with MASLD or NAFLD. The primary comparative outcome was the within-study difference in AUROC (ΔAUROC). The review also considered the validation setting, risk of bias, calibration, explainability, clinically relevant thresholds, and clinical utility to place comparative discrimination in a broader clinical context.

## Review

Methods

Study Design and Eligibility

This study was conducted as a diagnostic accuracy SR with an exploratory MA. It compared data-driven prediction models, including ML, AI, DL, radiomics, nomograms, and multivariable regression approaches, with conventional blood-based non-invasive tests for detecting advanced liver fibrosis in adults with MASLD or NAFLD. Reporting followed the Preferred Reporting Items for a Systematic Review and MA of Diagnostic Test Accuracy Studies (PRISMA-DTA) statement and PRISMA 2020 guidance [15,16]. The review was not prospectively registered in PROSPERO.

The primary clinical question was whether a prediction model provided greater diagnostic discrimination than an established blood-based fibrosis score when both were evaluated in the same patients for the same advanced fibrosis endpoint. The primary effect measure was the within-study difference in area under the AUROC. A positive ΔAUROC favoured the prediction model, whereas a negative value favoured the conventional blood-based test. The target condition was advanced liver fibrosis, defined as stage ≥F3 according to the fibrosis staging system used in the original study; reference standards included liver biopsy and, for secondary evidence, accepted non-invasive modalities such as elastography. This endpoint was selected because advanced fibrosis is directly relevant to risk stratification, referral decisions, and further diagnostic assessment.

Eligibility was defined using population, index model, comparator, target condition, reference standard, diagnostic outcome, and study design criteria. Studies using the earlier NAFLD or non-alcoholic steatohepatitis (NASH) terminology remained eligible when the study population was compatible with the contemporary MASLD disease spectrum and no other dominant liver disease defined the cohort.

To distinguish evidence according to its clinical strength, comparisons were classified into three evidence levels. Primary evidence required a separate validation cohort distinct from the model-development cohort, biopsy-defined ≥F3 fibrosis, and a same-cohort comparison with an eligible blood-based non-invasive test. Whether validation was conducted by the original model-development team or by an independent third party was recorded separately. Secondary evidence included internal holdout validation and/or an accepted non-invasive fibrosis reference such as elastography. Development, training, or discovery cohorts were retained for contextual interpretation but were not treated as primary validation evidence. The overall eligibility framework is summarised in Table 1.

**Table 1:** Eligibility Framework AI: artificial intelligence; APRI: aspartate aminotransferase-to-platelet ratio index; AUROC: area under the receiver operating characteristic curve; DL: deep learning; ELF: Enhanced Liver Fibrosis; FIB-4: Fibrosis-4 Index; MASH: metabolic dysfunction-associated steatohepatitis; MASLD: metabolic dysfunction-associated steatotic liver disease; ML: machine learning; NAFLD: non-alcoholic fatty liver disease; NASH: non-alcoholic steatohepatitis.

Component	Eligibility criteria
Population	Adults with MASLD, MASH, NAFLD, or NASH
Index model	ML, AI, DL, radiomics, nomogram, or multivariable regression models used for non-invasive fibrosis assessment
Comparator	Blood-based non-invasive tests, including FIB-4, NAFLD Fibrosis Score, APRI, and ELF
Target condition	Advanced liver fibrosis, defined as ≥F3
Reference standard	Liver biopsy or an accepted non-invasive reference, including elastography or magnetic resonance elastography, as reported by the original study
Outcomes	Test-specific AUROC and, where available, confidence intervals, sensitivity, specificity, or 2 × 2 diagnostic data
Study designs	Original diagnostic accuracy, cohort, cross-sectional, retrospective, prospective, development, or validation studies
Exclusions	Pediatric or animal studies; steatosis-only studies; studies without MASLD/NAFLD-specific data; reviews, editorials, letters, or guidelines without an original diagnostic cohort; studies without verifiable diagnostic-performance estimates

Imaging-based tests such as transient elastography and magnetic resonance elastography were retained as reference standards or for contextual interpretation where appropriate, but they were not pooled as blood-based comparators in the primary head-to-head analysis.

Information Sources and Search Strategy

The literature search was completed on June 1, 2026, using PubMed, Europe PMC, OpenAlex, and Semantic Scholar. Search concepts covered four domains: MASLD/NAFLD, advanced liver fibrosis, prediction modelling, and conventional non-invasive fibrosis assessment. Search terms included combinations of "MASLD", "MASH", "NAFLD", "NASH", “advanced fibrosis", "F3", “fibrosis staging", “artificial intelligence", “machine learning", “deep learning", “prediction model", "radiomics", "FIB-4", "APRI", “NAFLD Fibrosis Score", "ELF", “transient elastography", and “magnetic resonance elastography". PubMed and Europe PMC were searched using structured query families, whereas OpenAlex and Semantic Scholar were searched using 23 AI-focused and comparator-focused keyword queries.

No language restriction was applied during initial retrieval. Final eligibility required sufficient information to verify the study population, fibrosis endpoint, reference standard, comparator, cohort alignment, and diagnostic performance estimates. Reports that could not be retrieved or did not provide sufficient information for eligibility assessment were documented separately in the study-selection flow.

Because several overlapping query families were run within each information source, record management was performed in two stages. Duplicate hits generated within the same source were first consolidated before databases were merged. Cross-source duplicates were then identified using DOI and PMID matching, normalised bibliographic fields, and supplementary fuzzy matching of title, author, and publication year. The SR therefore distinguished raw query returns, records remaining after within-source consolidation, and records remaining after cross-source deduplication to preserve transparent PRISMA accounting. Complete source-specific search strings, query parameters, filters, search dates, and retrieval counts are provided in the Appendices.

Screening and Data Extraction

Title and abstract screening were automation-based. Two predefined rule-based eligibility profiles were applied to the deduplicated record set. The first used broader population, disease, and study design criteria, whereas the second applied stricter requirements related to fibrosis outcomes and extractable diagnostic performance information. A record retained by either profile was advanced for further consideration, while disagreements or uncertain classifications were conservatively assigned to a review category rather than automatically excluded.

This approach was used to support reproducible screening across a large evidence base and was not considered equivalent to independent screening by two human reviewers. Agreement between the two rule-based profiles was 94.7%. Their behaviour was initially evaluated using a stratified quality control (QC) sample of 328 records, comprising 100 likely includes, 100 uncertain records, and 128 likely excludes. Because a small non-zero inclusion rate was observed in the likely-exclude stratum, the exclusion process underwent additional validation before the final evidence lock.

A further independent sample of 500 records classified automatically as likely excluded was therefore assessed using a single-author reference standard. No eligible or potentially eligible studies were identified in this sample, corresponding to zero false negatives and a negative predictive value (NPV) of 100.0% (95% CI 99.2%-100.0%). The observed false-negative proportion was 0.0% (95% CI 0.0%-0.8%). These results were used to quantify the residual risk associated with automated exclusion rather than to claim equivalence with conventional dual-human screening.

The full-text eligibility assessment used structured rules supported by text parsing and bibliographic metadata. Records were classified as eligible for diagnostic extraction, relevant to narrative synthesis only, excluded, or not retrievable. Exclusion reasons included an ineligible population, an incorrect fibrosis endpoint, the absence of an appropriate reference standard, a lack of an eligible prediction model or comparator, the absence of extractable diagnostic-performance data, or publication type without an original diagnostic cohort. Study-level and test-level information was stored in a structured extraction database. The variables collected during data extraction are summarised in Table 2.

**Table 2:** Extracted Variables and Data Domains AUROC: Area under the receiver operating characteristic curve.

Domain	Extracted variables
Bibliographic information	Bibliographic identifiers, year, country, and study design
Sample characteristics	Sample size and numbers with and without advanced fibrosis
Population	Population characteristics and clinical setting
Fibrosis assessment	Fibrosis threshold and reference standard
Prediction model	Model type, input variables, and validation design
Comparator	Comparator name and comparator type
Diagnostic performance	AUROC, confidence intervals, sensitivity, and specificity
Validation	Development, internal holdout, separate-cohort, or third-party external validation status
Calibration and data handling	Calibration and missing-data reporting
Interpretability	Explainability or model-interpretability reporting
Clinical utility	Decision-curve or other clinical-utility assessment
Subgroup analysis	Subgroup evaluation
Model availability	Model, calculator, or code availability

Automated extraction from abstracts, structured XML, and available full texts was used to identify candidate diagnostic estimates efficiently. These outputs were treated only as extraction aids and did not establish eligibility for comparative synthesis. Candidate values subsequently underwent study-level verification to confirm the correct cohort, fibrosis threshold, reference standard, comparator identity, validation setting, and test-specific diagnostic estimates before they could contribute to the paired ΔAUROC analysis.

Verification of Paired Comparisons

The comparative synthesis was limited to original studies in which an eligible prediction model and a conventional blood-based fibrosis test were evaluated in the same patient cohort for advanced fibrosis (≥F3). This verification step was necessary because diagnostic estimates reported across different cohorts, endpoints, or reference standards cannot provide a valid head-to-head assessment of incremental performance.

A paired comparison was considered eligible only when the prediction model and blood-based comparator were evaluated in the same cohort; both estimates referred to the same ≥F3 endpoint; the same or a directly comparable reference standard was used; AUROC was clearly attributable to each named model or comparator; estimates represented original diagnostic data rather than values cited from another publication; and sufficient information was available to estimate ΔAUROC and its uncertainty. 

Comparisons were excluded when they addressed a different target condition, such as steatosis, MASH diagnosis, or portal hypertension; used comparator values from external publications; combined estimates from different cohorts or data splits; represented reviews or other non-original reports; or lacked verifiable test-specific performance estimates.

Where several eligible blood-based comparators were reported, FIB-4 was prioritised because it was the most clinically established comparator relevant to the primary question; NFS, aspartate aminotransferase-to-platelet ratio index (APRI), and enhanced liver fibrosis (ELF) remained eligible where applicable. Development and discovery results were not treated as equivalent to validation evidence. Separate-cohort biopsy-based validation constituted primary evidence, internal holdout or accepted non-invasive reference-standard evaluations constituted secondary evidence, and development cohorts were retained only for contextual interpretation.

Verification was performed at the cohort and test level using the source reports and bibliographic records. During this process, a reference-standard classification error identified in one eligible study prompted a targeted failure-mode audit of potentially affected full-text exclusions. Priority records identified through this audit were re-adjudicated using the same eligibility criteria before the evidence set was finalised. No study entered the MA solely on the basis of classification.

All final inclusion decisions, study identities, cohort roles, corrected values, and exclusion reasons were retained in an auditable decision record. The final verified evidence set was then frozen before statistical analysis, and all earlier provisionally paired datasets were treated as superseded.

Risk of Bias Assessment

Risk of bias was assessed only for studies contributing to the final verified comparative evidence. Quality Assessment of Diagnostic Accuracy Studies 2 (QUADAS-2) was used to evaluate patient selection, index test assessment, reference standard, and flow and timing [17]. Prediction model quality and applicability were additionally assessed using PROBAST+AI-informed domains, including participant selection, predictor definition, outcome assessment, model analysis, validation, calibration, and applicability [13]. Final judgements were based on the verified source studies rather than automated risk-of-bias classifications generated during earlier broad extraction. This ensured that quality assessment corresponded to the exact cohort and model evaluation contributing to the comparative synthesis.

Clinical-translation characteristics were presented separately from statistical discrimination. These included validation setting, whether validation was developer-led or performed by an independent third party, calibration, handling of missing data, clinically relevant thresholds, explainability, decision-curve assessment, subgroup assessment, and model availability. These features were used to assess whether improved discrimination was accompanied by evidence supporting practical clinical implementation. 

Statistical Analysis

The primary effect measure was the within-study difference in discrimination:

*&Delta;AUROC = AUROC (prediction model) &minus; AUROC (FIB-4)*

Positive values indicated greater discrimination by the prediction model. DerSimonian-Laird pooling [18] was retained only as a sensitivity approach and was not used as the principal basis for inference. Reported AUROC CIs were used to derive standard errors where available. When uncertainty estimates were not reported, AUROC variance was estimated using the Hanley-McNeil approach with the study-specific numbers of participants with and without advanced fibrosis [19,20]. No arbitrary default sample size was used. 

Because the two separate Sang validation cohorts arose from the same study and evaluated the same model, they were combined into a single study-level ΔAUROC using inverse-variance weighting before inclusion in the primary MA. This prevented one publication from receiving disproportionate weight. The individual validation cohorts were retained separately only for sensitivity analysis.

The principal synthesis used a random-effects model with between-study variance estimated by restricted maximum likelihood (REML). Hartung-Knapp adjustment was used for the 95% CI because the number of eligible studies was small and conventional normal-approximation intervals can be overly precise under such conditions. Between-study heterogeneity was summarised using I² but interpreted cautiously because of the limited number of studies. Because Hartung-Knapp intervals depend strongly on the number of meta-analytic units, CIs were expected to be especially wide when only two or three study-level effects were available. The two Sang validation cohorts were therefore combined at study level in the primary analysis to avoid giving a single model-development study disproportionate weight.

The primary MA was limited to separate-cohort biopsy-validation evidence. The secondary analysis additionally integrated eligible internal holdout evidence. Sensitivity analyses examined the effect of separating the two Sang validation cohorts and limiting the evidence to biopsy-based reference standards. 

Formal publication-bias assessment and meta-regression were not performed because the number of eligible studies was insufficient for reliable inference. Hierarchical summary receiver operating characteristic and bivariate diagnostic accuracy models were also not fitted because verified 2 × 2 data were unavailable in sufficient numbers. Sensitivity, specificity, likelihood ratios, and diagnostic odds ratios were therefore not pooled across studies. Because the included prediction models differed in architecture and inputs, the pooled ΔAUROC was treated as an exploratory summary of the direction and magnitude of model-specific ΔAUROC estimates relative to FIB-4 rather than as an estimate of a single clinically interchangeable prediction-model effect. All statistical analyses and evidence-management procedures were implemented in Python 3.11. Final analyses, tables, and figures were generated directly from the locked evidence dataset.

Results

Study Selection and Verification of Comparative Evidence

The four bibliographic sources returned 44,467 raw records across all search queries. After duplicate hits generated by overlapping queries were consolidated within each source, 25,287 records remained. A further 1,867 cross-source duplicates were removed, leaving 23,420 records for title and abstract screening. Of these, 2,941 reports were sought for retrieval. Forty-nine could not be retrieved, including 45 requiring unavailable institutional access and four that could not be obtained from the available sources. The remaining 2,892 reports underwent eligibility assessment. A total of 1,986 were excluded, while 290 were retained for diagnostic MA extraction and 616 for narrative context. The overall study-selection process, from record identification through eligibility assessment, is shown in Figure 1. 

**Figure 1:**
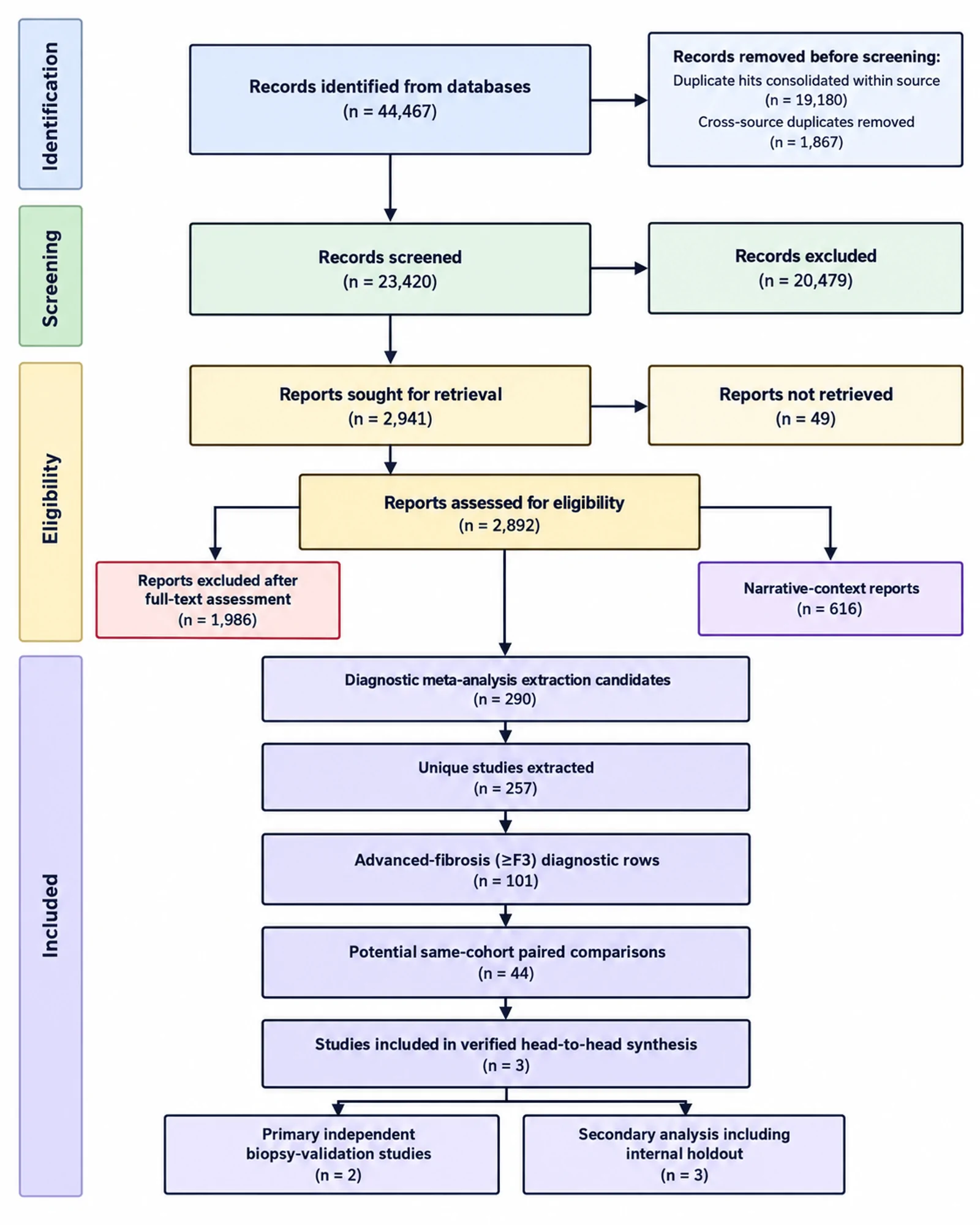
PRISMA 2020 Study Selection Flow Diagram PRISMA: Preferred Reporting Items for Systematic Reviews and Meta-Analyses

The 290 extraction candidates represented 257 unique studies. The advanced fibrosis dataset contained 101 diagnostic performance rows from 55 analytical study groups and 57 bibliographic records. Within this broad dataset, 44 potential same-cohort comparisons between a prediction model and a blood-based non-invasive test were identified.

Structured verification substantially reduced the number of comparisons suitable for head-to-head interpretation. The studies ultimately comprising the final verified comparative evidence were Sang et al. [21], Xiong et al. [22], and Okanoue et al. [23]. During final quality control, a reference standard classification error involving Okanoue et al. [23] led us to re-check a group of potentially affected full text exclusions. Twenty-six records were reviewed again using the same eligibility criteria. Twenty-five remained excluded after review, while that by Okanoue et al. [23] was confirmed as an eligible study with a separate biopsy-validation cohort. This also showed why automated screening alone was not sufficient, so all studies included in the final head-to-head analysis were checked again at the cohort and test level before statistical synthesis. Sang and Okanoue contributed developer-led separate-cohort biopsy-validation evidence to the primary analysis, whereas Xiong contributed internal holdout evidence to the secondary analysis. The additional verification and evidence-recovery process used to confirm the final head-to-head evidence set is shown in Figure 2. 

**Figure 2:**
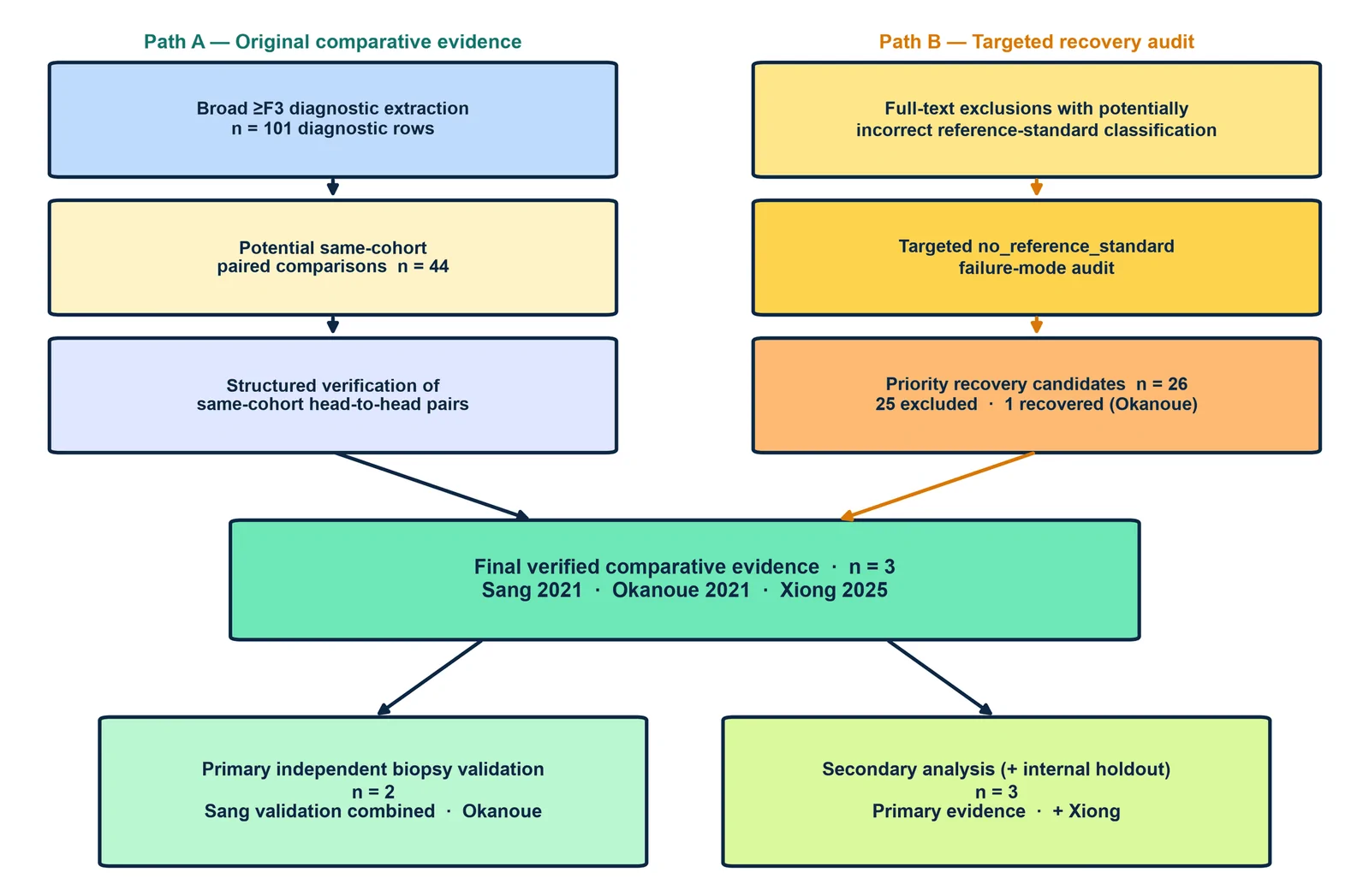
Verification and Evidence-Recovery Pathway

Additional validation of the automated exclusion process was performed in an additional sample of 500 records classified as likely exclude. No eligible or potentially eligible studies were identified, corresponding to zero false negatives and an NPV of 100.0% (95% CI 99.2%-100.0%). The estimated false-negative proportion was 0.0% (95% CI 0.0%-0.8%). The study-selection and final evidence-verification counts are summarised in Table 3.

**Table 3:** Study Selection and Final Evidence Verification

Selection stage	Number
Raw records across database queries	44,467
Records after within-source consolidation	25,287
Cross-source duplicates removed	1,867
Records screened after deduplication	23,420
Reports sought for retrieval	2,941
Reports not retrieved	49
Reports assessed for eligibility	2,892
Reports excluded after full-text assessment	1,986
Diagnostic meta-analysis extraction candidates	290
Narrative-context reports	616
Unique studies extracted	257
Advanced-fibrosis (≥F3) diagnostic rows	101
Analytical study groups / bibliographic records	55 / 57
Potential same-cohort paired comparisons	44
Priority recovery candidates adjudicated	26
Eligible study recovered through failure-mode audit	1
Final verified head-to-head studies	3
Primary separate-cohort biopsy-validation studies	2
Secondary analysis including internal holdout	3

Characteristics and Diagnostic Performance of the Included Studies

The final verified comparative evidence comprised three studies with 578 participants, including 173 patients with advanced fibrosis and 405 with stages F0-F2. The primary evidence consisted of two studies providing separate-cohort biopsy validation, involving 354 participants, while the secondary analysis additionally included the 224-participant internal holdout cohort reported by Xiong et al. [22].

Sang et al. [21] evaluated the logistic regression (LR)-based LiveFbr model in two separate validation cohorts. The first included 147 participants, of whom 31 had advanced fibrosis, and reported AUROCs of 0.89 for LiveFbr and 0.85 for FIB-4, giving a ΔAUROC of 0.040. The second validation cohort included 97 participants, of whom 32 had advanced fibrosis, and reported AUROCs of 0.71 and 0.63, respectively, giving a ΔAUROC of 0.080. Because both cohorts evaluated the same model within the same publication, their effects were combined at study level to avoid disproportionate weighting. The combined validation effect was ΔAUROC = 0.053 (95% CI -0.043 to 0.150). The original 540-participant discovery cohort was retained only as contextual development evidence and did not contribute to the primary synthesis.

Okanoue et al. [23] evaluated Fibro-Scope in a separate multicenter validation cohort of 110 biopsy-characterised patients, including 29 with F3-F4 fibrosis and 81 with F0-F2 fibrosis. Fibro-Scope achieved an AUROC of 0.911 compared with 0.771 for FIB-4, corresponding to a ΔAUROC of 0.140 (95% CI 0.017 to 0.263). This was the larger of the two separate-cohort estimates contributing to the primary analysis.

Xiong et al. [22] evaluated an XGBoost blood-panel model in an internal holdout cohort of 224 participants obtained through a 7:3 split of the original study population. The holdout sample included 81 patients with advanced fibrosis and 143 with F0-F2 fibrosis. The model achieved an AUROC of 0.917 compared with 0.752 for FIB-4, producing a ΔAUROC of 0.165 (95% CI 0.091 to 0.239). Because this estimate arose from internal rather than separate-cohort validation, it was included in the secondary analysis rather than the strict primary evidence set. All three study-level ΔAUROC point estimates were positive, although the evidence base was small and the estimates arose from different validation settings. Importantly, all three studies were evaluated by the original model-development teams. Sang et al. [21] and Okanoue et al. [23] used separate validation cohorts, whereas Xiong et al. [22] used an internal holdout cohort. None of the models had been independently validated by a third-party group. The separate-cohort results should therefore be interpreted cautiously because developer-led validation may produce more optimistic estimates than fully independent external validation. Study characteristics and head-to-head diagnostic estimates are summarised in Table 4.

**Table 4:** Characteristics and Paired Diagnostic Performance of the Final Verified Studies AUROC: area under the receiver operating characteristic curve; CI: confidence interval; FIB-4: Fibrosis-4 Index; LR: logistic regression.

Study	Prediction model	Validation evidence	Reference standard	Sample, N (F3+ / F0-F2)	Model AUROC	FIB-4 AUROC	ΔAUROC (95% CI)	Analysis role
Sang et al. [21]	LR LiveFbr	Two separate validation cohorts within the same study, combined at study level	Biopsy	244 (63 / 181)	0.89/0.71	0.85/0.63	0.053 (-0.043 to 0.150), combined	Primary
Okanoue et al. [23]	Fibro-Scope	Separate multicenter validation cohort within the developer study	Biopsy	110 (29 / 81)	0.911	0.771	0.140 (0.017 to 0.263)	Primary
Xiong et al. [22]	XGBoost blood-panel model	Internal holdout validation	Biopsy	224 (81 / 143)	0.917	0.752	0.165 (0.091 to 0.239)	Secondary

Risk of Bias and Clinical-Translation Characteristics

All three final comparative studies were judged to have an overall unclear risk of bias using QUADAS-2. Patient selection and reference-standard domains were rated as low risk across all studies. The index-test domain was unclear for Sang et al. [21], Okanoue et al. [23], and Xiong et al. [22], reflecting incomplete reporting or uncertainty around aspects of test specification and assessment. Flow and timing were rated as low risk for Sang et al. [21] and unclear for Okanoue et al. [23] and Xiong et al. [22]. The domain-level judgements are shown in Figure 3.

**Figure 3:**
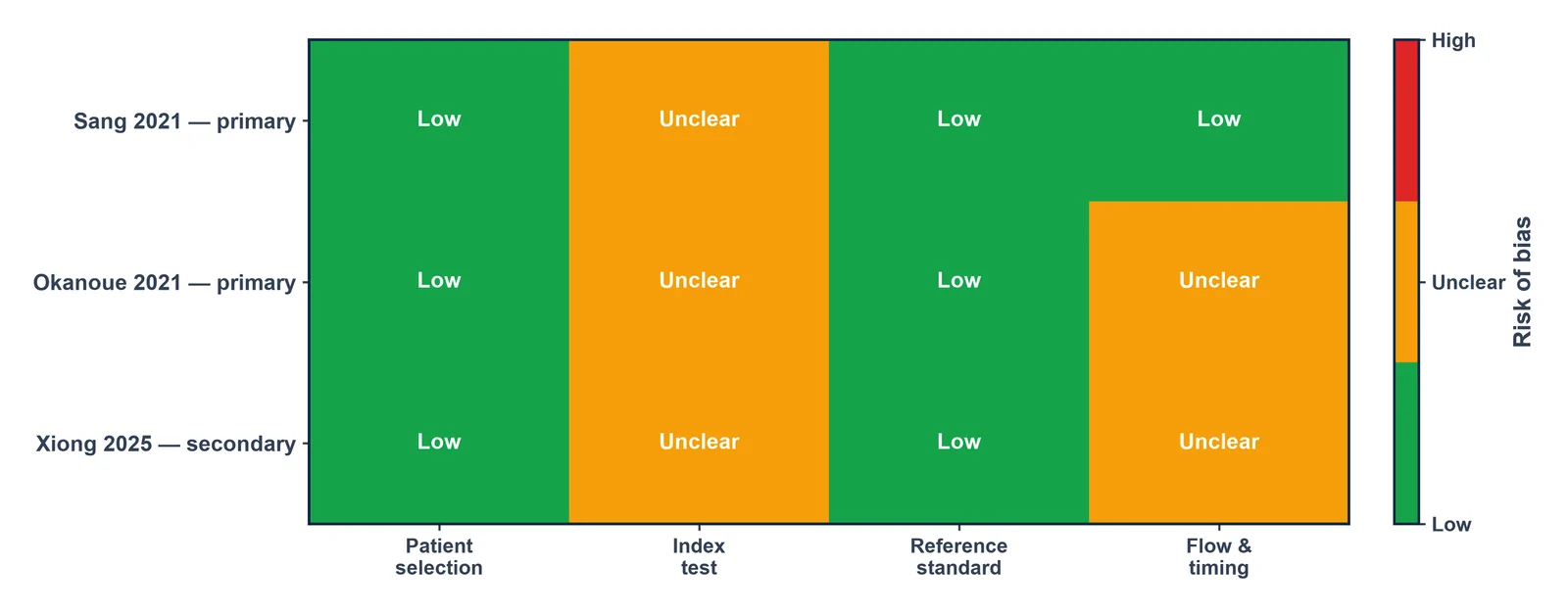
QUADAS-2 Risk-of-Bias Assessment of the Final Included Evidence QUADAS-2 domain-level risk-of-bias assessment for Sang et al. [21], Okanoue et al. [23], and Xiong et al. [22]. QUADAS-2: Quality Assessment of Diagnostic Accuracy Studies 2.

The studies also differed in validation design. Sang et al. [21] and Okanoue et al. [23] provided separate-cohort validation and therefore contributed to the primary evidence tier, whereas Xiong et al. [22] used an internal holdout design and contributed to the secondary tier. This distinction was retained throughout the quantitative synthesis rather than treating development, internal validation, and separate-cohort validation as equivalent forms of evidence.

Clinical-translation reporting remained less complete than discrimination reporting. Across the final evidence set, AUROC-based comparative performance was consistently available, but calibration, decision-curve analysis, clinically actionable thresholds, implementation resources, and other measures of real-world utility were reported inconsistently. Comparative discrimination was reported more consistently than calibration or clinical-utility measures, but the small evidence base limited firm conclusions about either diagnostic advantage or readiness for clinical implementation. The QUADAS-2 assessments and validation status of the included studies are summarised in Table 5.

**Table 5:** QUADAS-2 Assessment and Validation Status of the Final Included Studies QUADAS-2: Quality Assessment of Diagnostic Accuracy Studies 2.

Study	Patient selection	Index test	Reference standard	Flow and timing	Overall QUADAS-2	Validation tier
Sang et al. [21]	Low	Unclear	Low	Low	Unclear	Primary
Okanoue et al. [23]	Low	Unclear	Low	Unclear	Unclear	Primary
Xiong et al. [22]	Low	Unclear	Low	Unclear	Unclear	Secondary

Head-to-Head Comparative Performance and Exploratory Meta-Analysis

The three verified studies yielded positive ΔAUROC point estimates, but the evidence base was small and all evaluations were conducted by the original model-development teams. The combined separate-cohort estimate from Sang et al. [21] was ΔAUROC = 0.053 (95% CI -0.043 to 0.150), while Okanoue et al. [23] reported a separate-cohort estimate of 0.140 (95% CI 0.017 to 0.263). Xiong et al. [22], which contributed internal holdout evidence, reported ΔAUROC = 0.165 (95% CI 0.091 to 0.239). Because these were different prediction models, the individual estimates were interpreted separately and the pooled analysis was considered exploratory.

The primary MA was limited to the two studies providing separate-cohort biopsy-validation evidence, Sang et al. [21] and Okanoue et al. [23]. Using a random-effects model with REML and Hartung-Knapp adjustment, the pooled ΔAUROC was 0.086 (95% CI -0.448 to 0.621). The CI was very wide and included no difference, reflecting the substantial uncertainty associated with a two-study evidence base. 

The secondary analysis additionally integrated the internal holdout cohort from Xiong et al. [22]. The pooled ΔAUROC increased to 0.126 (95% CI -0.023 to 0.276). Although the CI was narrower and the point estimate remained positive, it still included no difference. The principal and secondary analyses are shown in Figure 4A and Figure 4B, respectively.

**Figure 4:**
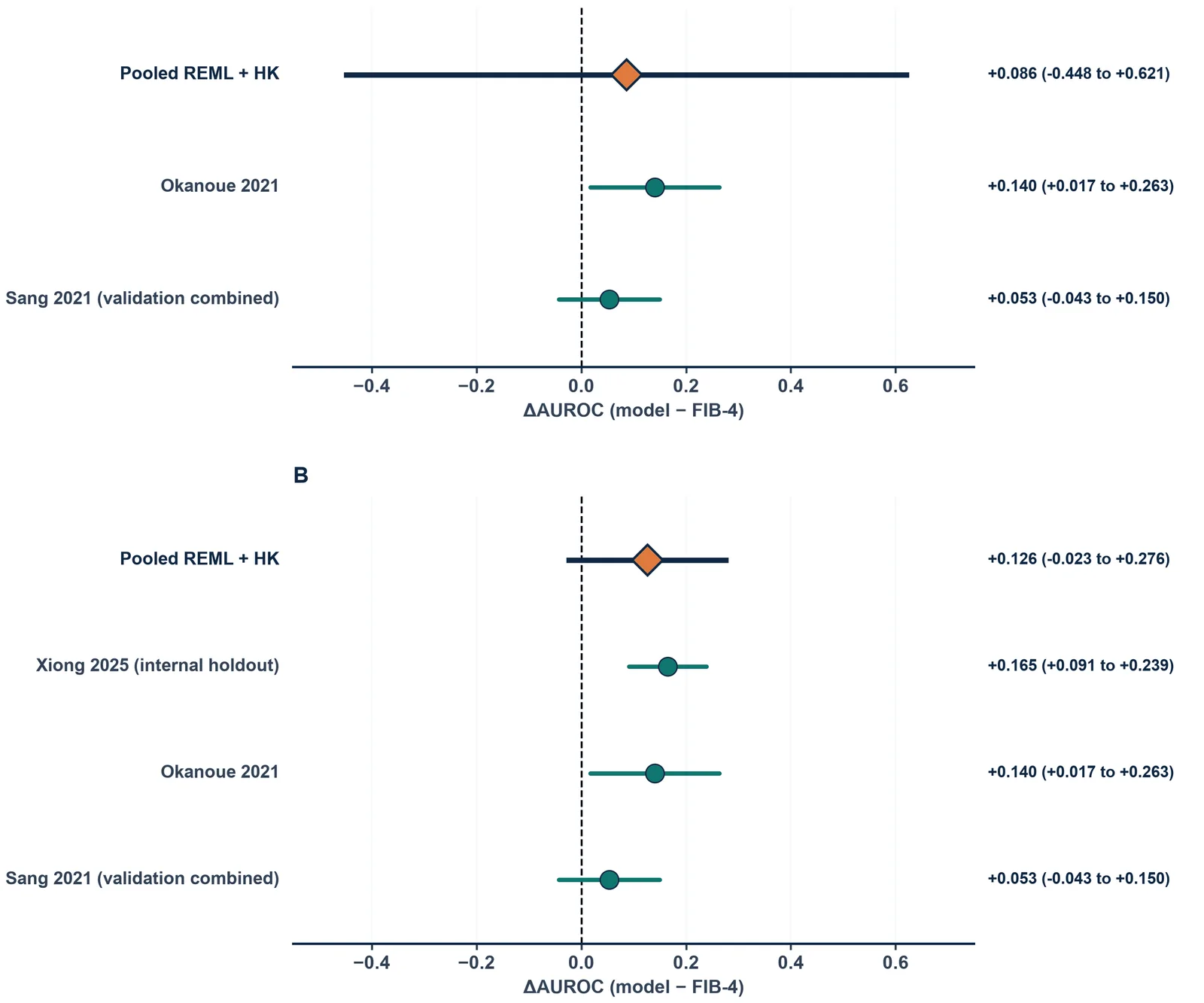
Exploratory Pooled Comparison of Prediction Models Versus FIB-4 for Advanced Fibrosis Study estimates are from Sang et al. [21], Okanoue et al. [23], and Xiong et al. [22]. Panel A shows the primary analysis restricted to separate-cohort biopsy-validation evidence. Panel B shows the secondary analysis after inclusion of the internal holdout validation cohort. ΔAUROC: difference in area under the receiver operating characteristic curve; CI: confidence interval; FIB-4: Fibrosis-4 Index.

A sensitivity analysis treated the two separate Sang validation cohorts as separate estimates rather than combining them at study level. With these two cohorts, Okanoue et al. [23], and Xiong et al. [22], the pooled ΔAUROC was 0.127 (95% CI 0.034 to 0.219). This analysis produced a narrower CI. However, it was retained as a sensitivity analysis because treating two cohorts from the same publication as separate MA units could give disproportionate influence to a single model-development study. The study-level combined estimate therefore remained the preferred representation for the primary analysis.

The difference in CI width across analyses reflects both the small number of meta-analytic units and the Hartung-Knapp adjustment. The primary analysis contained only two study-level effects, whereas the secondary analysis contained three and the sensitivity analysis four. Treating the two Sang cohorts separately increased the effective number of units and produced a narrower interval, but these cohorts originated from the same model-development study and were therefore not considered fully independent observations. The study-level analysis remained the preferred analysis.

The biopsy-only sensitivity analysis produced the same pooled estimate as the broader three-study analysis because all final comparative cohorts used biopsy-defined fibrosis. DerSimonian-Laird estimates were retained as sensitivity outputs only and were not used as the principal basis for inference. The primary, secondary, and sensitivity analyses are summarised in Table 6.

**Table 6:** Final Pooled Comparative Analyses CI: confidence interval; ΔAUROC: difference in area under the receiver operating characteristic curve.

Analysis	k	Pooled ΔAUROC	Hartung-Knapp 95% CI	Interpretation
Primary: independent biopsy-validation evidence	2	0.086	-0.448 to 0.621	Principal analysis
Secondary: Primary: separate-cohort biopsy-validation evidence	3	0.126	-0.023 to 0.276	Broader validation evidence
Biopsy-only sensitivity	3	0.126	-0.023 to 0.276	Same three verified biopsy cohorts
Sang validation cohorts analyzed separately + Okanoue + Xiong	4	0.127	0.034 to 0.219	Sensitivity analysis only

All three study-level point estimates were positive, but the strength of inference remained limited by the small number of studies, developer-led validation, and wide pooled CIs. The primary analysis was especially imprecise with only two separate-cohort studies, while the secondary analysis also remained compatible with no difference. The sensitivity analysis produced a narrower CI when the two Sang cohorts were treated separately, but this approach could give disproportionate influence to a single model-development study. The pooled results should therefore be regarded as exploratory rather than evidence of a generalisable advantage over FIB-4. Unpaired AUROC summaries from the broader extraction dataset were retained only as supplementary descriptive context and were not used for comparative inference.

Overall, the current evidence does not establish that prediction models outperform FIB-4. The positive study-level estimates warrant further evaluation, but third-party external validation, calibration, clinically meaningful thresholds, and evidence of clinical utility are needed before any advantage can be considered reproducible or clinically actionable.

Discussion

Principal Findings and Interpretation

The available head-to-head evidence was too limited and imprecise to establish that data-driven prediction models outperform FIB-4. Three studies contributed verified comparisons, and all were conducted by the original model-development teams. Sang et al. [21] and Okanoue et al. [23] provided separate-cohort biopsy-validation evidence and formed the primary analysis, whereas Xiong et al. [22] contributed internal holdout evidence to the secondary analysis. The study-level ΔAUROC point estimates were 0.053 for Sang et al. [21], 0.140 for Okanoue et al. [23], and 0.165 for Xiong et al. [22]. Although all three point estimates were positive, the models differed in architecture and inputs and should not be interpreted as a clinically interchangeable class. Their individual estimates therefore remain more informative than the exploratory pooled average.

The primary pooled ΔAUROC was 0.086, with a wide Hartung-Knapp 95% CI of -0.448 to 0.621. The interval included no difference and reflected the substantial uncertainty associated with only two separate-cohort studies. Adding the internal holdout evidence from Xiong et al. [22] produced a pooled ΔAUROC of 0.126 (95% CI -0.023 to 0.276), which also remained compatible with no difference. The pooled analyses should therefore be interpreted as exploratory summaries rather than evidence of a stable or generalisable advantage over FIB-4.

Clinical and Real-World Implications

FIB-4 remains an important first-line tool because it is inexpensive, transparent, and based on routinely available clinical and laboratory variables. It can be deployed at scale without specialist software or additional testing. Its known limitations, including indeterminate classifications and variable performance across patient groups and settings, nevertheless create an opportunity for more flexible prediction models.

The positive study-level estimates justify further evaluation of these models, but an increase in AUROC alone would not be sufficient to justify clinical implementation. A model with better discrimination should also improve decisions at clinically relevant thresholds, provide reliable individual risk estimates, generalise to new populations, and add value to existing diagnostic pathways. Evidence for calibration, decision-curve analysis, net benefit, implementation feasibility, and workflow impact remained limited across the verified studies. The evidence is more informative about discrimination than clinical utility but remains insufficient to establish either a reliable diagnostic advantage or readiness for routine implementation. 

The most useful role for prediction models may therefore not be direct replacement of FIB-4. A more realistic pathway would be to evaluate them as adjunctive or second-stage tools, particularly for patients with indeterminate or discordant first-line results. FIB-4 could retain its role as an accessible initial screen, while a validated prediction model could help determine which patients require elastography, specialist assessment, or further investigation. Although the separate-cohort validation reported by Sang et al. [21] and Okanoue et al. [23] shows that positive incremental performance is not confined entirely to model-development data, two independent studies remain insufficient to define where such models should be placed in routine care. Prospective pathway-based evaluation is therefore needed before their use can be recommended as part of standard fibrosis assessment.

Comparison With Previous Evidence and Methodological Contribution

Previous reviews have reported high or promising diagnostic performance for AI and ML approaches in MASLD-related and NAFLD-related fibrosis [11,12]. Those findings are not necessarily inconsistent with the present review. Most previous syntheses have focused primarily on how accurately prediction models discriminate between fibrosis stages, whereas the present study addressed a narrower clinical question: whether a prediction model provides additional discrimination over FIB-4 when both are tested in the same patients for the same advanced fibrosis endpoint.

This distinction matters because AUROCs obtained from separate studies are not directly comparable. Differences in disease prevalence, patient selection, referral setting, fibrosis spectrum, reference standard, and validation design can substantially affect reported performance. A model with a high AUROC in one cohort cannot therefore be assumed to outperform FIB-4 evaluated in another. Same-cohort comparison reduces these sources of between-study confounding and more closely reflects the decision faced in clinical practice.

A further contribution of this review was the explicit separation of development, internal validation, and separate-cohort validation evidence. The original Sang discovery cohort was retained for contextual interpretation but was not treated as primary evidence. Its two separate validation cohorts were instead combined into a single study-level effect, while the internal holdout result from Xiong et al. [22] was analyzed separately. The review therefore distinguished development cohorts, internal holdout validation, developer-led separate-cohort validation, and third-party external validation; no third-party external validation was identified. 

The verification process also identified an important evidence-recovery issue. A reference-standard classification error affecting Okanoue et al. [23] prompted a targeted audit of potentially affected full-text exclusions. Twenty-six priority candidates were reassessed; 25 remained excluded after scientific review, while Okanoue was recovered and subsequently verified as an eligible study with a separate multicenter biopsy-validation cohort. This audit illustrates that large diagnostic literatures can contain many apparently relevant comparisons, but only a much smaller subset may support valid head-to-head inference once cohort alignment, target condition, reference standard, comparator identity, validation status, and attributable performance values are verified.

The screening process was also evaluated quantitatively rather than being presented as equivalent to conventional dual-human review. In a single-author reference assessment of 500 automatically excluded records, no eligible or potentially eligible studies were identified, giving an NPV of 100.0% (95% CI 99.2%-100.0%) and an observed false-negative proportion of 0.0% (95% CI 0.0%-0.8%). This does not replace independent human screening, but it provides a measured estimate of residual exclusion error rather than relying only on agreement between automated screening profiles.

Limitations and Future Research Priorities

Several limitations should be considered when interpreting these findings. First, only two studies contributed to the primary analysis, resulting in a very wide Hartung-Knapp CI and limiting the precision of the pooled estimate. All three included evaluations were conducted by the original model-development teams, and no third-party external validation was identified, creating a risk of optimistic performance estimates. The included prediction models also differed substantially in architecture, inputs, populations, and validation design, so the pooled ΔAUROC should be regarded only as an exploratory summary rather than a common class effect. 

The synthesis also relied mainly on AUROC because sufficiently complete 2 × 2 diagnostic data were unavailable. Future studies should report clinically actionable thresholds, sensitivity, specificity, predictive values, calibration, and decision-curve or net-benefit analyses. Verified comparative evidence was sufficient only for FIB-4, preventing reliable conclusions for NFS, APRI, ELF, or other non-invasive tests; future evaluations should therefore compare prediction models directly with multiple established clinical comparators where appropriate.

Screening was automation-assisted rather than conventional dual-human screening, 49 reports were not retrieved, and several major bibliographic databases were not searched. Although the 500-record validation sample identified no false negatives, some residual risk of missed evidence remains. In addition, reporting of calibration, missing-data handling, subgroup performance, model availability, implementation cost, and clinical utility was inconsistent. Future studies should therefore prioritise independent third-party, multicenter external validation using the same ≥F3 endpoint and direct same-cohort comparison with FIB-4 across diverse clinical settings and patient populations. 

Finally, the review was not prospectively registered, although methodological changes, verification decisions, and the final evidence lock were documented. Future reviews should use prospective registration and predefined analytical plans to further strengthen transparency and reproducibility. The present evidence supports further evaluation of data-driven prediction models as potential complements to FIB-4 rather than immediate replacements. The central unanswered question is whether the positive study-level estimates can be reproduced in independent third-party populations and translated into better clinical decisions.

## Conclusions

This verification-audited systematic review found that the available head-to-head evidence is too limited and imprecise to establish that data-driven prediction models outperform FIB-4 for advanced fibrosis. All three study-level point estimates favoured the prediction models, but the primary pooled estimate had a wide confidence interval that included no difference, and the secondary pooled estimate also remained compatible with no difference. Two studies evaluated their models in separate biopsy-validation cohorts, while a third used an internal holdout cohort; all evaluations were conducted by the original model-development teams rather than by independent third-party investigators. The findings therefore should be considered preliminary and do not support replacing FIB-4. Future studies should prioritise third-party multicentre validation, calibration, clinically meaningful thresholds, decision-curve or net-benefit assessment, and direct evaluation of effects on referral, elastography, biopsy, and patient management. Progress in MASLD fibrosis prediction will depend less on producing additional high-AUROC models and more on demonstrating reproducible added value over established clinical pathways. 

## Appendices

Search sources and date

The literature search was conducted on June 1, 2026, across PubMed, Europe PMC, OpenAlex, and Semantic Scholar. PubMed was accessed through the NCBI E-utilities interface, Europe PMC through its REST API, OpenAlex through the Works API, and Semantic Scholar through the Graph API. No language restriction was applied during retrieval. The final search accounting included 44,467 raw source returns, which were reduced to 25,287 records after within-source consolidation and 23,420 records after cross-source deduplication.

**Table 7:** Sources

Source	Search interface	Search date	Raw records in final search accounting
PubMed	NCBI E-utilities (esearch and efetch)	June 1, 2026	3,346
Europe PMC	REST API	June 1, 2026	29,101
OpenAlex	Works API	June 1, 2026	11,520
Semantic Scholar	Graph API	June 1, 2026	500
Total			44,467

PubMed and Europe PMC search strategy

PubMed and Europe PMC were searched using the same three Boolean query families. These queries were designed to capture studies addressing MASLD/NAFLD fibrosis prediction, conventional non-invasive fibrosis tests, and diagnostic prediction models.

" ( MASLD OR MASH OR NAFLD OR NASH OR "metabolic dysfunction-associated steatotic liver disease" OR "metabolic dysfunction-associated steatohepatitis" OR "non-alcoholic fatty liver disease" OR "nonalcoholic fatty liver disease" OR "non-alcoholic steatohepatitis" OR "nonalcoholic steatohepatitis" ) AND ( fibrosis OR "advanced fibrosis" OR "significant fibrosis" OR cirrhosis OR "liver fibrosis" OR "fibrosis staging" ) AND ( "artificial intelligence" OR "machine learning" OR "deep learning" OR radiomics OR "neural network" OR "random forest" OR XGBoost OR "support vector machine" OR "computer-aided diagnosis" OR "prediction model" ) "

Query 2: Conventional non-invasive fibrosis tests

"( MASLD OR MASH OR NAFLD OR NASH OR "non-alcoholic fatty liver disease" OR "nonalcoholic fatty liver disease" ) AND ( fibrosis OR "advanced fibrosis" OR cirrhosis ) AND ( "FIB-4" OR APRI OR "NAFLD fibrosis score" OR NFS OR ELF OR elastography OR FibroScan OR "magnetic resonance elastography" OR MRE ) AND ( "diagnostic accuracy" OR sensitivity OR specificity OR AUROC OR AUC )  "

Query 3: Diagnostic prediction models

" (NAFLD OR NASH OR MASLD OR MASH) AND ("advanced fibrosis" OR "significant fibrosis" OR "fibrosis staging") AND ("prediction model" OR "diagnostic accuracy" OR AUROC OR sensitivity OR specificity) "

The query level retrieval counts recorded in the search log are shown below.

**Table 8:** Numbers of records

Search query	PubMed	Europe PMC
AI and MASLD/NAFLD fibrosis	551	8,695
Conventional non-invasive fibrosis tests	1,694	11,149
Diagnostic prediction models	1,102	9,257
Europe PMC total		29,101

For PubMed, searches used db=pubmed, JSON output for search results, and XML for record retrieval. PubMed results were retrieved using retmax=10000 with sequential retstart values, followed by record retrieval in batches of 200. No date, language, or manually specified sort restriction was applied. Europe PMC searches used format=json, resultType=core, and cursor-based pagination with a page size of 1,000 records. Retrieval continued until all available pages had been obtained, with no language or publication-date restriction.

OpenAlex and Semantic Scholar keyword searches

OpenAlex and Semantic Scholar were searched using the same 23 keyword strings. These searches were intended to broaden retrieval beyond the structured PubMed and Europe PMC queries and capture both AI-focused and conventional comparator-focused studies.

**Table 9:** Keywords NAFLD: non-alcoholic fatty liver disease; MASLD: metabolic dysfunction-associated steatotic liver disease; MASH: metabolic dysfunction-associated steatohepatitis

No.	Exact keyword query
1	NAFLD fibrosis machine learning
2	NAFLD fibrosis artificial intelligence
3	MASLD fibrosis machine learning
4	MASH fibrosis deep learning
5	NASH fibrosis radiomics
6	liver fibrosis NAFLD XGBoost
7	liver fibrosis NAFLD random forest
8	liver fibrosis NAFLD neural network
9	NAFLD fibrosis radiomics MRI
10	NAFLD fibrosis ultrasound deep learning
11	NAFLD fibrosis elastography machine learning
12	MASLD fibrosis radiomics
13	MASH fibrosis MRI artificial intelligence
14	NAFLD FIB-4 advanced fibrosis diagnostic accuracy
15	NAFLD APRI fibrosis sensitivity specificity
16	NAFLD fibrosis score advanced fibrosis AUROC
17	MASLD FIB-4 fibrosis staging
18	NAFLD transient elastography advanced fibrosis
19	NAFLD magnetic resonance elastography fibrosis staging
20	NAFLD fibrosis prediction model
21	MASLD fibrosis prediction model
22	NASH advanced fibrosis prediction model
23	noninvasive fibrosis prediction NAFLD

OpenAlex search parameters and retrieval

OpenAlex searches used plain-text search= queries with the filters from_publication_date:2010-01-01 and type:article. Results were retrieved using cursor pagination with 200 records per page. A configured retrieval stop was applied once approximately 500 records had been accumulated for a query, which resulted in an observed maximum of 600 records for several high-yield searches.

**Table 10:** OpenAlex Search Parameters NAFLD: non-alcoholic fatty liver disease; MASLD: metabolic dysfunction-associated steatotic liver disease; MASH: metabolic dysfunction-associated steatohepatitis

OpenAlex query	Records retrieved
NAFLD fibrosis machine learning	600
NAFLD fibrosis artificial intelligence	600
MASLD fibrosis machine learning	600
MASH fibrosis deep learning	419
NASH fibrosis radiomics	234
liver fibrosis NAFLD XGBoost	248
liver fibrosis NAFLD random forest	600
liver fibrosis NAFLD neural network	600
NAFLD fibrosis radiomics MRI	197
NAFLD fibrosis ultrasound deep learning	600
NAFLD fibrosis elastography machine learning	600
MASLD fibrosis radiomics	94
MASH fibrosis MRI artificial intelligence	181
NAFLD FIB-4 advanced fibrosis diagnostic accuracy	600
NAFLD APRI fibrosis sensitivity specificity	600
NAFLD fibrosis score advanced fibrosis AUROC	600
MASLD FIB-4 fibrosis staging	547
NAFLD transient elastography advanced fibrosis	600
NAFLD magnetic resonance elastography fibrosis staging	600
NAFLD fibrosis prediction model	600
MASLD fibrosis prediction model	600
NASH advanced fibrosis prediction model	600
noninvasive fibrosis prediction NAFLD	600
Total	11,520

Semantic Scholar search parameters and retrieval

Semantic Scholar searches used limit=100 with offset-based pagination and a configured maximum of 1,000 records per query. Requested fields included title, abstract, year, authors, external identifiers, journal, citation count, URL, publication type, and open-access information. No date or language restriction was applied. Semantic Scholar retrieval was affected by API connectivity and rate-limiting problems. Five searches returned 100 records each, while the remaining 18 searches failed after retry attempts and were recorded as unreachable.

**Table 11:** Semantic Scholar Search Parameters and Retrieval NAFLD: non-alcoholic fatty liver disease; MASLD: metabolic dysfunction-associated steatotic liver disease; MASH: metabolic dysfunction-associated steatohepatitis

Semantic Scholar query	Records retrieved
NAFLD fibrosis radiomics MRI	100
MASH fibrosis MRI artificial intelligence	100
NAFLD transient elastography advanced fibrosis	100
NAFLD fibrosis prediction model	100
MASLD fibrosis prediction model	100
Remaining 18 searches	0
Total	500

API requests were rate-limited and allowed up to three retry attempts. HTTP 429 responses triggered exponential backoff. These retrieval limitations were retained in the reporting rather than treated as complete coverage of the respective databases.

Record consolidation and deduplication

Because the same publication could be retrieved by several query families or databases, records were consolidated in two stages. First, records sharing the same DOI, PMID, OpenAlex identifier, or Semantic Scholar identifier were merged while retaining the richest available metadata and source-query information. This reduced the raw retrieval pool from 44,467 source returns to 25,287 records. Cross-source deduplication was then performed using exact DOI, PMID, PMCID, OpenAlex, and Semantic Scholar identifiers, followed by normalized-title matching and fuzzy title matching using a token-sort similarity threshold of 0.92. A further 1,867 duplicates were removed, leaving 23,420 unique records for title and abstract screening.

## References

[1] Rinella ME, Lazarus JV, Ratziu V (2023). A multisociety Delphi consensus statement on new fatty liver disease nomenclature. J Hepatol.

[2] Riazi K, Azhari H, Charette JH (2022). The prevalence and incidence of NAFLD worldwide: a systematic review and meta-analysis. Lancet Gastroenterol Hepatol.

[3] Younossi ZM, Golabi P, Paik JM, Henry A, Van Dongen C, Henry L (2023). The global epidemiology of nonalcoholic fatty liver disease (NAFLD) and nonalcoholic steatohepatitis (NASH): a systematic review. Hepatology.

[4] Dulai PS, Singh S, Patel J (2017). Increased risk of mortality by fibrosis stage in nonalcoholic fatty liver disease: systematic review and meta-analysis. Hepatology.

[5] Ekstedt M, Hagström H, Nasr P, Fredrikson M, Stål P, Kechagias S, Hultcrantz R (2015). Fibrosis stage is the strongest predictor for disease-specific mortality in NAFLD after up to 33 years of follow-up. Hepatology.

[6] Sterling RK, Lissen E, Clumeck N (2006). Development of a simple noninvasive index to predict significant fibrosis in patients with HIV/HCV coinfection. Hepatology.

[7] Angulo P, Hui JM, Marchesini G (2007). The NAFLD fibrosis score: a noninvasive system that identifies liver fibrosis in patients with NAFLD. Hepatology.

[8] (2024). EASL-EASD-EASO Clinical Practice Guidelines on the management of metabolic dysfunction-associated steatotic liver disease (MASLD). J Hepatol.

[9] Patel K, Sebastiani G (2020). Limitations of non-invasive tests for assessment of liver fibrosis. JHEP Rep.

[10] Castera L, Friedrich-Rust M, Loomba R (2019). Noninvasive assessment of liver disease in patients with nonalcoholic fatty liver disease. Gastroenterology.

[11] Decharatanachart P, Chaiteerakij R, Tiyarattanachai T, Treeprasertsuk S (2021). Application of artificial intelligence in non-alcoholic fatty liver disease and liver fibrosis: a systematic review and meta-analysis. Ther Adv Gastroenterol.

[12] Souza NS, Vitório TC, Souza RA, Machado MA, Cotrim HP (2026). Artificial intelligence for fibrosis diagnosis in metabolic-dysfunction-associated steatotic liver disease: a systematic review. Diagnostics (Basel).

[13] Moons KG, Damen JA, Kaul T (2025). PROBAST+AI: an updated quality, risk of bias, and applicability assessment tool for prediction models using regression or artificial intelligence methods. BMJ.

[14] Collins GS, Dhiman P, Ma J (2024). Evaluation of clinical prediction models (part 1): from development to external validation. BMJ.

[15] McInnes MD, Moher D, Thombs BD (2018). Preferred Reporting Items for a Systematic Review and Meta-analysis of Diagnostic Test Accuracy studies: the PRISMA-DTA statement. JAMA.

[16] Page MJ, McKenzie JE, Bossuyt PM (2021). The PRISMA 2020 statement: an updated guideline for reporting systematic reviews. BMJ.

[17] Whiting PF, Rutjes AW, Westwood ME (2011). QUADAS-2: a revised tool for the quality assessment of diagnostic accuracy studies. Ann Intern Med.

[18] DerSimonian R, Laird N (1986). Meta-analysis in clinical trials. Control Clin Trials.

[19] Hanley JA, McNeil BJ (1982). The meaning and use of the area under a receiver operating characteristic (ROC) curve. Radiology.

[20] Hanley JA, McNeil BJ (1983). A method of comparing the areas under receiver operating characteristic curves derived from the same cases. Radiology.

[21] Sang C, Yan H, Chan WK (2021). Diagnosis of fibrosis using blood markers and logistic regression in Southeast Asian patients with non-alcoholic fatty liver disease. Front Med (Lausanne).

[22] Xiong FX, Sun L, Zhang XJ (2025). Machine learning-based models for advanced fibrosis in non-alcoholic steatohepatitis patients: A cohort study. World J Gastroenterol.

[23] Okanoue T, Shima T, Mitsumoto Y (2021). Novel artificial intelligent/neural network system for staging of nonalcoholic steatohepatitis. Hepatol Res.

